# *Msh2* depletion impairs cell differentiation, proliferation and metabolism in esophageal organoids

**DOI:** 10.1242/jcs.264470

**Published:** 2026-10-19

**Authors:** Marjorie Rolland, Dominique Jean, Maude Hamilton, Molly Sedeuil, Alexis Gonneaud, Line Berthiaume, Étienne Audet-Walsh, Véronique Giroux

**Affiliations:** ^1^ Department of Immunology and Cell Biology, Faculty of Medicine and Health Sciences, Université de Sherbrooke, Sherbrooke, J1E 4K8, Canada; ^2^ Centre de Recherche du Centre Hospitalier de l'Université de Sherbrooke (CRCHUS), Sherbrooke, J1H 5N4, Canada; ^3^ Institut de Recherche sur le Cancer de l'Université de Sherbrooke (IRCUS), Sherbrooke, J1E 4K8, Canada; ^4^ Department of Molecular Medicine, Faculty of Medicine, Université Laval, Québec, G1V 0A6, Canada; ^5^ Endocrinology – Nephrology Axis, CHU de Québec-Université Laval Research Center, Québec, G1V 4G2, Canada

**Keywords:** Esophageal organoid, *Msh2*, Clonogenicity, Differentiation, Metabolism

## Abstract

Given its rapid cell turnover, the esophageal stratified squamous epithelium must maintain homeostasis. This process is ensured by a long-lived subpopulation of basal stem cells that express keratin 15 (*Krt15*). However, the mechanisms that specifically control their characteristics, namely, self-renewal, multipotency and regeneration, remain unknown. Interestingly, MutS homolog 2 (*Msh2*), implicated in the DNA mismatch repair pathway, is the most upregulated gene in *Krt15-*positive esophageal stem cells compared to *Krt15*-negative cells. Accentuated DNA repair capacity is often observed in stem cells to maintain their genomic integrity and avoid the acquisition of mutations. Hence, this study aimed to evaluate the effects of *Msh2* loss on stem cell functions, including differentiation, clonogenicity and proliferation, in murine esophageal organoids. *Msh2*-depleted organoids were generated by CRISPR/Cas9. They displayed morphogenic alterations and significant homeostasis perturbations. Cell differentiation was decreased while clonogenicity and proliferation were increased. Loss of *Msh2* also caused lower expression of stem cell markers and changes in response to chemotherapy drugs. Proteomic analysis confirmed variations in epithelial cell differentiation and showed major changes in proteins associated with metabolic processes. In line with these results, metabolomic analysis confirmed an altered abundance of various metabolites of the tricarboxylic acid cycle and biosynthetic pathways. In conclusion, these results showed that *Msh2* is implicated in the homeostasis of esophageal organoids, potentially through metabolic rewiring.

## Teaser

**Summary:** Msh2, a protein involved in mismatch repair, maintains characteristics associated with stem cells, like the regulation of the proliferation–differentiation balance and response to stress in esophageal organoids.

## INTRODUCTION

To ensure survival, every organism, tissue, and cell needs to maintain homeostasis. The esophagus, composed of a stratified squamous epithelium, is no exception. Esophageal epithelial equilibrium is controlled by signaling pathways such as bone morphogenic proteins (BMPs), Wingless-int1 (Wnt)/β-catenin and Notch, which are also involved in esophageal morphogenesis and development (Rosekrans et al., 2015). The esophageal epithelium renews every 7 to 10 days in mice. This renewal is ensured by the basal cells that migrate towards the lumen while undergoing differentiation into suprabasal cells to reach the final stage of keratinized cells. Heterogeneity in the esophageal basal cell layer has been shown using organotypic culture (Kalabis et al., 2008) and organoids (DeWard et al., 2014). Furthermore, the use of a lineage tracing method confirmed the presence of a long-lived subpopulation of basal cells marked by keratin 15 (*Krt15*) in the mouse esophageal epithelium (Giroux et al., 2017). Interestingly, *KRT15* expression has been confirmed in a quiescent cell population in the human esophagus (Busslinger et al., 2021; Rochman et al., 2022). These cells displayed many stem cell properties, such as self-renewal, multipotency, proliferation, tissue regeneration and resistance to high-dose radiation (Giroux et al., 2017).

In between stem cell and differentiated cell states lie progenitor cells, which usually have a lower self-renewal capacity than stem cells but a higher proliferation rate (Blanpain et al., 2007). What allows stem and progenitor cells to maintain tissue integrity despite their high cell turnover and to display resistance to several DNA-damaging agents is their enhanced capacity to repair DNA (Blanpain et al., 2011). To prevent genomic instability, different DNA repair mechanisms will be used depending on stem cell type, but also context. For example, embryonic stem cells display a strong homologous recombination (HR) (Yoon et al., 2014; Choi et al., 2018), whereas trachea basal stem cells favor non-homologous end joining (NHEJ) (Weeden et al., 2017). The importance of DNA repair proteins in the maintenance of genomic stability is also stem cell type-dependent, as shown by Zhang and collaborators. Indeed, exonuclease I (Exo1) has been shown to be essential for the self-renewal and proliferation of neuronal stem cells but not of hematopoietic stem cells (Zhang et al., 2014). As expected, RNA sequencing showed that esophageal *Krt15-*positive (*Krt15*+) stem cells are significantly enriched for genes associated with DNA integrity compared to *Krt15*-negative (*Krt15*−) cells. Still, the promoted DNA repair mechanism is currently unknown in these cells. Interestingly, the most upregulated gene in *Krt15*+ esophageal cells is MutS homolog 2 (*Msh2*) (600 times higher than in *Krt15−* cells) (Giroux et al., 2017).

Msh2 is part of the DNA damage recognition in mismatch repair (MMR) and is well known for being part of the genomic integrity machinery. The MutSα complex (Msh2–Msh6) recognizes mismatches and small indels (1 to 3 nucleotides) whereas the MutSβ complex (Msh2–Msh3) recognizes bigger indels (1 to 15 nucleotides) (Hsieh and Zhang, 2017). The heterodimer formation is necessary to allow Msh2 activity (Johnson et al., 1996), as confirmed by many studies showing that Msh2 depletion also leads to Msh6 loss of expression (Franck et al., 2021; Lin et al., 2014; Rath et al., 2019; Madden-Hennessey et al., 2022). Other DNA alterations, such as cyclobutane pyrimidine dimers (Liu et al., 2017), O^6^-methylguanine (Yoshioka et al., 2006) and 8-oxo-2′-deoxyguanosine (Colussi et al., 2002), are also detected by MutS dimers, supporting the implication of Msh2 in other DNA damage responses.

Although the role of Msh2 remains unknown in esophageal stem cells, several studies suggest that this protein could play an important role in other stem cells. In fact, induction of pluripotency in fibroblasts leads to a higher expression of MMR proteins compared to that in parental fibroblasts (Lin et al., 2014; Ku et al., 2010; Roos et al., 2007). Msh2 importance in lymphoma development has also been studied in hematopoietic stem cells (Qing and Gerson, 2017). In the gastrointestinal tract, two studies showed that Msh2 was more expressed in proliferating and less differentiated intestinal cells than in differentiated cells (Wilson et al., 1995; Tomé et al., 2013), suggesting a potential role of Msh2 in the maintenance of intestinal stem cells. Even if these studies suggest changes in MMR proteins expression in stem cells, the specific role of Msh2 in this distinct cell type remains elusive. Interestingly, Keysselt and collaborators have shown that complete loss of *Msh2* in mouse intestinal organoids leads to microsatellite instability (Keysselt et al., 2017), demonstrating the importance of Msh2 in genomic integrity. In fact, Msh2 mutations occur in many cancer types, such as colorectal, endometrial, or gastric cancers, confirming its crucial role in the maintenance of genome stability (Dowty et al., 2013). For example, Lynch syndrome, also known as hereditary non-polyposis colorectal cancer, is caused by pathogenic variants in MMR genes and is associated with a higher risk of developing colorectal cancer but also other cancer types.

As mentioned above, *Msh2* is the most overexpressed gene in *Krt15*+ stem cells compared to *Krt15*− cells (Giroux et al., 2017). Although Msh2 is known to have a role in the maintenance of many stem cell types, the promoted DNA repair mechanism in esophageal stem cells is currently unknown. Thereby, we propose that *Msh2* could be essential in the maintenance of the esophageal epithelium and stem cell functions. Herein, we established *Msh2*-depleted esophageal organoids using CRISPR/Cas9 and determined the effects of *Msh2* loss on clonogenicity, proliferation, multipotency and regeneration.

## RESULTS

### Loss of *Msh2* causes morphological changes correlating with altered differentiation in esophageal organoids

To investigate the role of Msh2 in esophageal epithelial homeostasis, we established mouse esophageal organoids depleted for *Msh2* using a CRISPR/Cas9 approach. First, the loss of *Msh2* was confirmed by western blotting and immunofluorescence (Fig. 1A,B). In other studies using CRISPR or short hairpin RNA (Franck et al., 2021; Lin et al., 2014; Rath et al., 2019; Madden-Hennessey et al., 2022), loss of Msh2 also leads to a massive diminution in the expression of its obligatory partner Msh6, as shown in Fig. 1A. As Msh2 is known to be implicated in the maintenance of genomic integrity, the nuclear shape of the cells forming the organoids, which can be used to monitor overall genomic integrity, was analyzed. Lamin B was used to mark the nuclear envelope. The nuclear circularity of *Msh2*-depleted cells was significantly reduced as determined from the form factor, confirming the validity of our model (Fig. 1C).

**Fig. 1.**
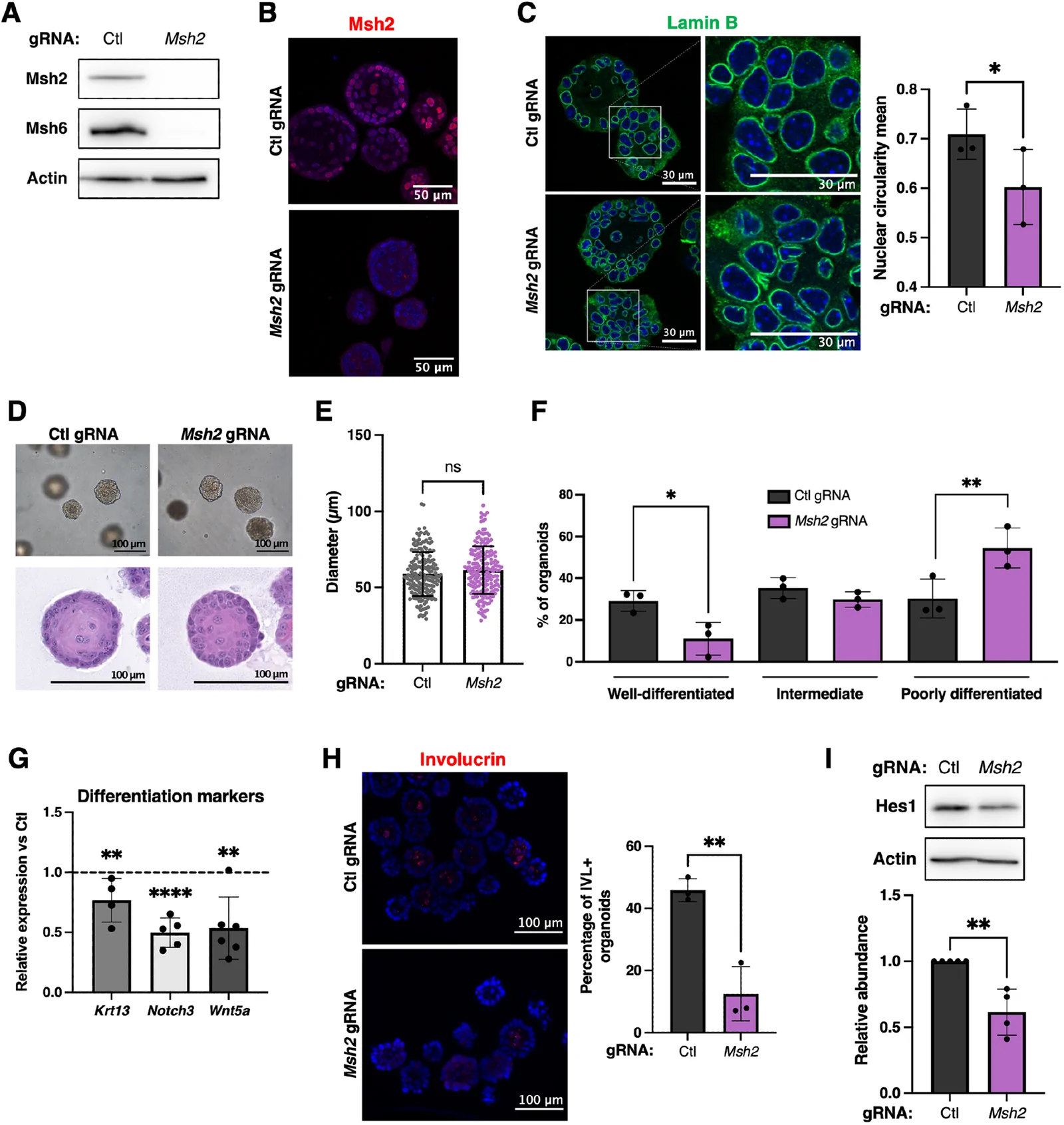
***Msh2* loss causes morphological changes correlated with altered differentiation in esophageal organoids.** (A–I) *Msh2* was depleted in mouse esophageal organoids using a CRISPR/Cas9 approach. (A) Western blot for Msh2 and Msh6 was done in both populations. Actin was used as the loading control (*n*=5 biological replicates). (B) Immunofluorescence for Msh2 (in red) was performed in control and in *Msh2-*depleted organoids (*n*=2 biological replicates). DAPI was used as a nuclear stain. (C) Immunofluorescence of the nuclear membrane marker lamin B was performed and DAPI was used to stain nuclei. Graph shows the nuclear circularity calculated with the form factor using Cell Profiler. Bar graph shows the mean±s.d. (*n*=3 biological replicates, count of 53–83 nuclei/condition/replicate). (D) Representative images of organoids in brightfield and H&E coloration are presented. (E) Diameter of organoids was measured from the H&E coloration. Each dot represents the diameter of an organoid. Bar graph shows mean±s.d. (*n*=4 biological replicates, count of 50 organoids/condition/replicate). (F) Organoids were scored for differentiation status from H&E coloration by two individuals who were not aware of the experimental conditions. Bar graph shows mean±s.d. (*n*=3 biological replicates, count of a minimum of 28 organoids/condition/replicate). (G) Relative expression of differentiation genes normalized to *Gapdh* (Ctl) was measured by qPCR in both populations. Bar graph shows the mean±s.d. (*n*=4–6 biological replicates). (H) Immunofluorescence of the terminal differentiation marker involucrin was done and DAPI was used to stain nuclei. Graph shows the percentage of IVL+ organoids. Bar graph shows the mean±s.d. (*n*=3 biological replicates, count of 21–56 organoids/condition/replicate). (I) Western blot for Hes1, a Notch pathway target, was performed in *Msh2*-depleted and control organoids. Actin was used as a loading control. Bar graph shows the mean±s.d. (*n*=4 biological replicates). *P<0.05; **P<0.01; ns, not significant (unpaired two-tailed Student's *t*-test). Ctl, control.

Interestingly, *Msh2* loss led to significant changes in the morphology of organoids. Under a brightfield microscope, fewer organoids presented a black keratinized center in the *Msh2*-depleted organoid population versus controls (Fig. 1D, upper panels). These macroscopic observations were confirmed histologically. Esophageal organoids typically comprise a well-organized outer basal cell layer and a keratinized center (Deward et al., 2014). However, *Msh2*-depleted organoids displayed a disorganized basal cell layer and less keratinization, suggesting an imbalance in the basal-to-suprabasal cell ratio (Fig. 1D, lower panels). To better characterize these modifications, the diameter of organoids was measured, but no significant changes were observed (Fig. 1E). However, a higher proportion of DAPI+ cells per area was observed in *Msh2*-depleted organoids (Fig. S1B), confirming a possible accumulation of basal cells, as they are smaller than suprabasal cells. Because of the altered organoid morphology observed in the absence of *Msh2* (Fig. 1D), the differentiation levels were compared. We established a scoring system that classifies organoids as well differentiated, intermediately differentiated and poorly differentiated (Fig. S1A). As expected, controls are well distributed between each phenotype, whereas there is a switch from well-differentiated to poorly differentiated for *Msh2*-depleted organoids (Fig. 1F).

To confirm the attenuated pattern of differentiation, the expression of differentiation-specific genes was quantified by quantitative PCR (qPCR). A previous single-cell RNA sequencing study showed that *Krt13*, *Notch3* and *Wnt5a* are overexpressed in esophageal differentiated cells (Busslinger et al., 2021). These genes were significantly decreased in the absence of *Msh2* (Fig. 1G). Next, the number of organoids stained with the terminal differentiation marker involucrin (IVL) was evaluated; the percentage of IVL+ organoids was 3.7-fold lower in the absence of *Msh2* (Fig. 1H). These results suggest a reduction in differentiation in *Msh2*-depleted organoids. Knowing that the Notch pathway greatly participates in human esophageal epithelial differentiation (Ohashi et al., 2010), we evaluated the protein level of the Notch target gene, *Hes1*. Interestingly, the loss of *Msh2* significantly reduced Hes1 expression (Fig. 1I), suggesting a possible downregulation of the Notch pathway. Downregulation of the Notch pathway is typically associated with a loss of differentiation and basal cell hyperplasia as we observed (Zhang et al., 2018).

### Depletion of *Msh2* in esophageal organoids increases clonogenicity but decreases the expression of stem cell markers

As differentiation of organoids is altered by *Msh2* loss and tissue homeostasis is mainly orchestrated by stem cells, we next investigated self-renewal capacity, one of the distinct features of stem cells. Hence, the organoid formation rate (OFR; i.e. the proportion of cells in a population capable of forming an organoid) was evaluated. Contrary to what was expected, *Msh2* loss caused a ∼60% increase in the ability to form organoids (Fig. 2A). Using the same method, organoids were subjected to chemotherapeutic drugs, because it is known that stem cells typically maintain enhanced clonogenic potential even following injury and also display resistance to anti-cancer treatments. In fact, *Msh2*-depleted organoids were less sensitive to the OFR reduction induced by 5-fluorouracil (5-FU) and cisplatin (Fig. 2B), suggesting that loss of Msh2 increases clonogenicity even upon treatment with these chemotherapeutic drugs. However, *Msh2* loss did not alter the effects of camptothecin and etoposide, which, respectively, inhibit topoisomerase I and II (Tilsed et al., 2022), on clonogenicity (Fig. 2B). As the properties of stem cells are affected by *Msh2* absence, the expression of stem cell markers was quantified. qPCR analysis showed a significant decrease in the expression of genes linked to stemness like *CD73*, *Smoc2*, *Lrig1* and *Prom2* (Fig. 2C). *Lrig* regulates quiescent stem cells, *Prom2* is associated with cancer stem cells and, *Smoc2* is involved in the maintenance of intestinal stem cells. Because *CD73* is one of the first stem cell markers described in the esophageal epithelium (DeWard et al., 2014), we validated this marker by flow cytometry. Indeed, the percentage of CD73+ cells was significantly decreased by almost 50% in the absence of *Msh2* (Fig. 2D). In short, along with reduced differentiation, *Msh2* loss was associated with reduced expression of stemness markers while surprisingly enhancing clonogenicity. We, therefore, hypothesized that *Msh2*-depleted organoids could be enriched with cells displaying a phenotype similar to progenitor cells.

**Fig. 2.**
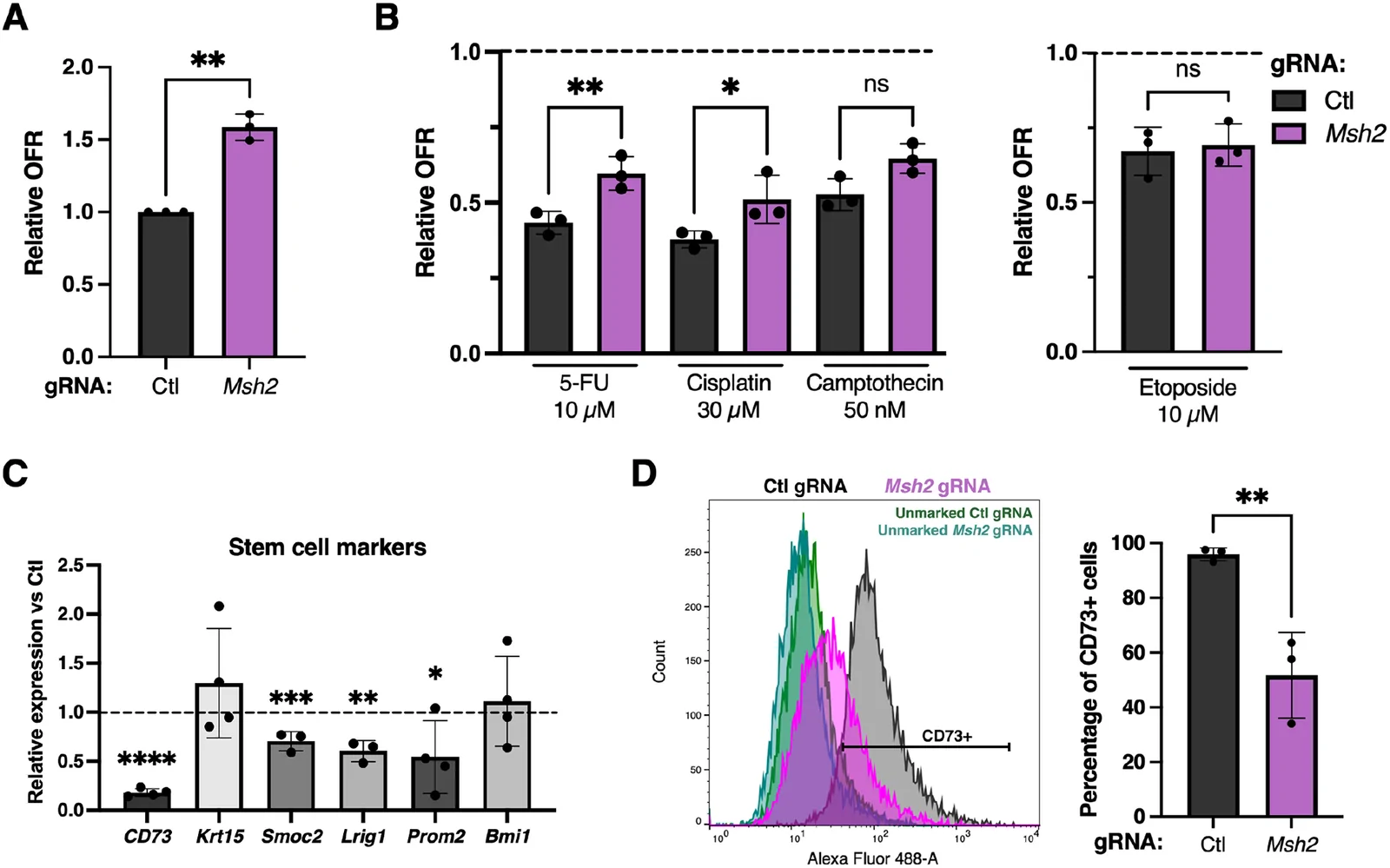
***Msh2*-depleted organoids display higher clonogenicity but lower stem cell marker expression.** (A) Organoids were seeded at 300 cells per well. The OFR was measured 6 days post seeding. Bar graph shows the mean±s.d. (*n*=3 biological replicates). (B) Organoids were treated with chemotherapeutic drugs [5-FU (5-fluorouracil), cisplatin, camptothecin and etoposide] at day 1 for 72 h. The OFR was calculated 6 days post seeding. Bar graph shows the mean±s.d. (*n*=3 biological replicates) normalized to relative the OFR of vehicle (DMF or DMSO) illustrated by the dotted lines. (C) Relative expression of genes associated with stem cells was measured by qPCR in *Msh2*-depleted organoids compared to controls. *Gapdh* (Ctl) was used as a reference gene. Bar graph shows the mean±s.d. (*n*=4–6 biological replicates). (D) CD73+ cells were quantified by flow cytometry in both populations. Unmarked controls are in blue for *Msh2* gRNA and in green for Ctl gRNA. Bar graph shows the mean±s.d. (*n*=3 biological replicates, count of 10,000 events/replicate. *P<0.05; **P<0.01; ***P<0.001; ****P<0.0001; ns, not significant (unpaired two-tailed Student's *t*-test). Ctl, control.

### Cell proliferation is increased in *Msh2*-depleted organoids

Given that progenitor cells are known to be highly proliferative and *Msh2*-depleted organoids accumulate basal cells, which are also typically proliferative cells, we next performed WST-1 assays, which assess cell viability by measuring cellular metabolic activity. Growth curves showed that *Msh2*-depleted organoids were significantly more proliferative from day 3 and up to day 7 post-seeding (Fig. 3A). Confirming the increased proliferation rate, a higher number of Ki67+ cells was observed in *Msh2*-depleted organoids (Fig. 3B). Moreover, enhanced levels of proliferation marker phospho-histone H3^S10^ were also noticed in *Msh2*-depleted organoids by western blotting (Fig. 3C). Levels of phospho-β-catenin^Ser552^ were also increased in the absence of *Msh2* (Fig. 3D). When phosphorylated on serine 552, β-catenin accumulates, thus promoting its transcriptional activity (He et al., 2007; Fang et al., 2007; Taurin et al., 2006). This result, therefore, suggests an activation of the Wnt/β-catenin pathway, which is essential for the proliferation of stem cells of several epithelia (Van Camp et al., 2014). We also confirmed that these observations were not due to changes in viability nor in cell cycle progression (Fig. S2). In short, clonogenic and proliferative capacities of esophageal organoids are enhanced in the absence of *Msh2*, whereas differentiation is decreased, suggesting an imbalance in tissue homeostasis.

**Fig. 3.**
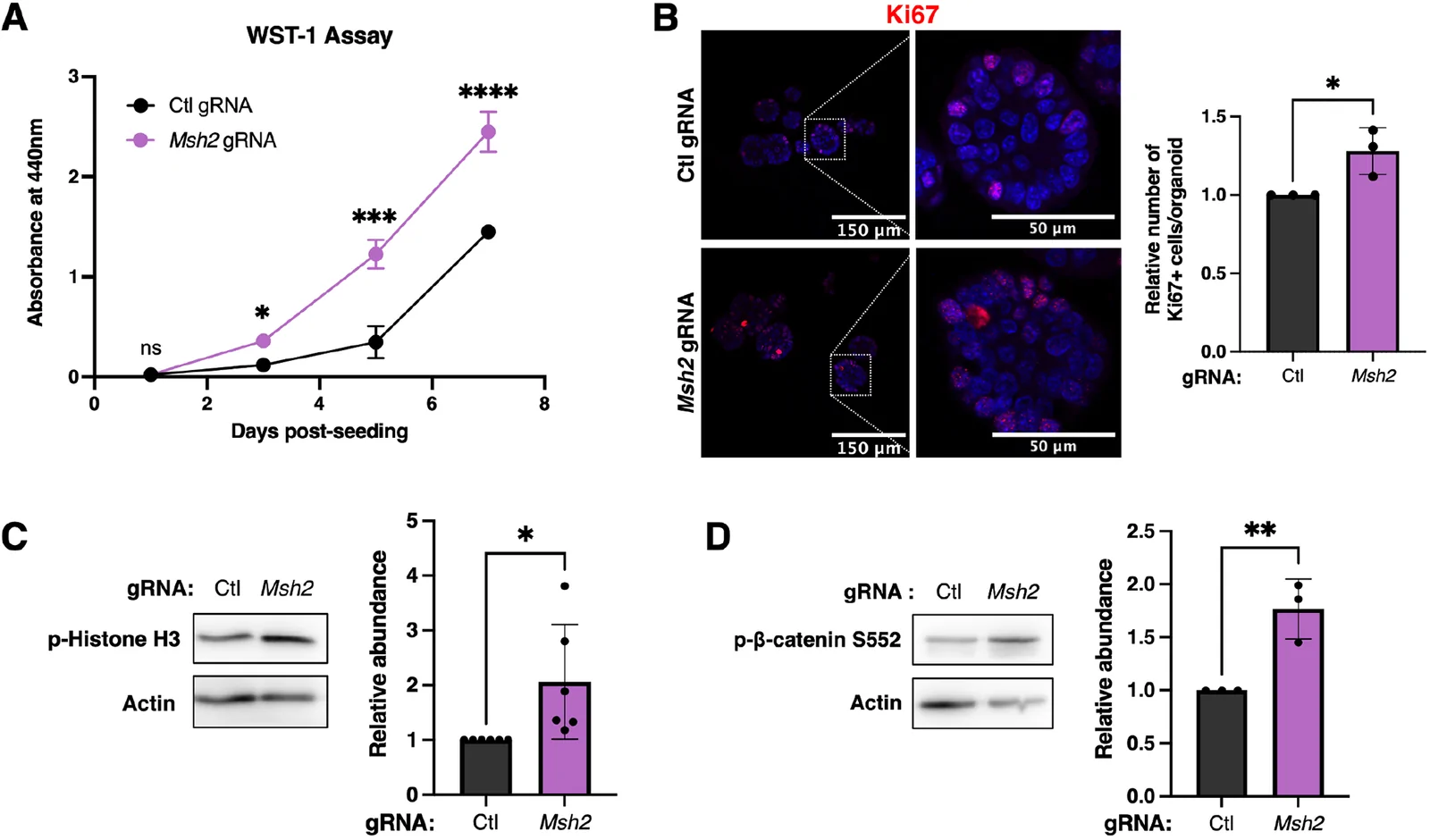
***Msh2* depletion increases proliferation in esophageal organoids.** (A) Representative growth curve for each organoid population was obtained using a WST-1 assay. Bar graph shows the mean±s.d. (*n*=5 biological replicates). (B) Immunofluorescence of the proliferation marker Ki67. Nuclei were stained with DAPI. Graph shows the relative number of Ki67+ cells per organoid. Bar graph shows mean±s.d. (*n*=3 biological replicates, count of 17–34 organoids/condition/replicate). (C,D) Western blot for phospho-histone H3^S10^ (C; *n*=6 biological replicates) and for phospho-β-catenin^S552^ (D; *n*=4 biological replicates) were performed using actin as a loading control. Bar graph shows the mean±s.d. *P<0.05; **P<0.01; ***P<0.001; ****P<0.0001; ns, not significant (unpaired two-tailed Student's *t*-test). Ctl, control.

### Proteomic profiling suggests major changes in metabolism in *Msh2*-depleted organoids

To better understand the molecular mechanisms underlying the functional differences observed between *Msh2*-depleted and control organoids, a shotgun mass spectrometry analysis was performed. Proteins were considered as differentially expressed when detection intensity was altered by a log2 fold-change (FC) higher than 1.5 and a statistical significance lower than 0.05 (Advaita Bio's iPathwayGuide). A total of 163 differentially expressed proteins were identified (Table S1). In these proteins, several were associated with biological processes relevant to the phenotypes we observed, such as epithelial cell differentiation (Rock2, Flnb and Sprr1a), DNA repair (Trp53bp1, Ssrp1 and Exd2) or Wnt signaling pathway (Cdh1, Cav1 and Ccar2), as illustrated in Fig. 4A. This supports that cell differentiation and proliferation are affected by *Msh2* loss. Some of those proteins were also present in the top 20 upregulated and downregulated proteins (Fig. 4B). Numerous proteins associated with cell adhesion were also significantly altered in *Msh2*-depleted versus control organoids (Cdh1, Cldn4, Capn1, Lgals1, Lgals3 and Lgals7). Gene ontology (GO) terms and biological processes analysis were used to highlight the pathways that were significantly enriched in *Msh2*-depleted organoids. Interestingly, several metabolic processes were highlighted as the top GO terms (Fig. 4C), suggesting a possible metabolic rewiring of *Msh2*-depleted organoids. For example, GO terms that were enriched include small molecular metabolic process, organophosphate metabolic process, carbohydrate derivative metabolic process and lipid metabolic process. Various proteins implicated in those GO metabolic processes were identified as significantly changed by *Msh2* loss (Fig. 4D). For example, 5′-nucleotidase, cytosolic II (Nt5c2), a hydrolase involved in purine metabolism and glutamate-pyruvate transaminase 1 (Gpt), an enzyme facilitating the generation of pyruvate and glutamate were upregulated, whereas carbonic anhydrase 2 (Ca2), an enzyme allowing the hydration of carbon dioxide in bicarbonate and thus, controlling the cell pH, was downregulated in *Msh2*-depleted organoids.

**Fig. 4.**
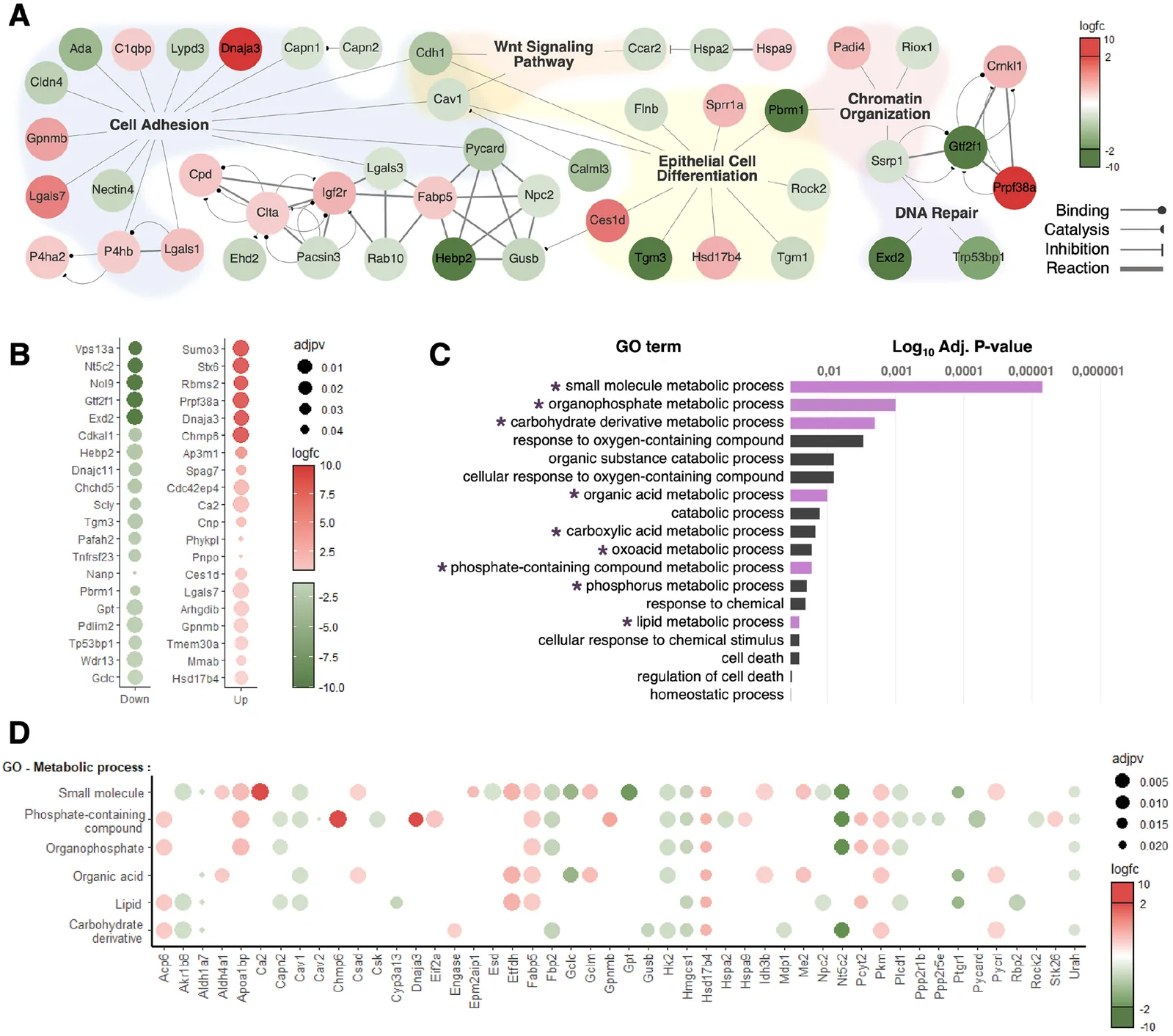
**Proteomic profiling demonstrates changes in several proteins associated with cell metabolism in *Msh2*-depleted organoids.** (A–D) Mass spectrometry was performed on total protein extracts of control (Ctl) and *Msh2*-depleted organoids (*n*=3 biological replicates). The results were analyzed using Advaita Bio's iPathwayGuide. Upregulated proteins are shown in red and downregulated proteins are in green. (A) Network showing significantly regulated proteins connected to relevant biological processes is illustrated. (B) The 20 most upregulated and downregulated proteins are shown. Dot size represents adjusted *P*-value and color, log2 fold change (FC) compared to the control. (C) GO terms with a minimum of 20 differentially expressed genes significantly changed are represented. Graph illustrates log10 adjusted p-value (adjusted *P*-value). Stars show GO terms related to metabolic processes. (D) Proteins differentially expressed in GOs related to metabolic processes are illustrated. Dot size represents adjusted *P*-value (with a cut-off at 0.025) and color, log2 FC compared to control.

### Metabolomic analysis confirms changes in several metabolites associated with the TCA cycle and amino acid metabolism in *Msh2*-depleted organoids

As many proteins involved in various metabolic processes were shown to be dysregulated in proteomic analysis, we performed metabolomic analysis on *Msh2*-depleted and control organoids. Detected metabolites are illustrated using a heatmap (Fig. 5A) and show a global alteration of metabolite levels in *Msh2-*depleted organoids. Most significantly altered metabolites were downregulated, suggesting a slower metabolic rate. Several Kyoto encyclopedia of genes and genomes (KEGG)-enriched metabolite sets were also significantly dysregulated (Fig. 5B), including key energetic pathways as well as amino acid pathways. Notably, several metabolites connected to the TCA cycle were reduced. These included succinate and malate, but also closely related metabolites, such as aspartate, that can be synthesized from oxaloacetate (OAA; Fig. 5C). A trend in the increase of glutamine, an amino acid that can also fuel the TCA cycle, was observed, as well as a significant increase in glycerol, which can be freed when cells use triglycerides as a source of energy to fuel fatty acid β-oxidation. Other significantly decreased metabolites included additional amino acids, such as glycine and methionine, which are both crucial for nucleotide synthesis through one-carbon metabolism (Fig. 5D). In line with this, the nucleobase uracil was significantly decreased by 32% (Fig. 5D). Given that *Msh2*-depleted organoids exhibited increased cell proliferation (Fig. 3), a global decrease in metabolite pools could indicate increased pressure on biosynthetic pathways required to sustain this proliferation.

**Fig. 5.**
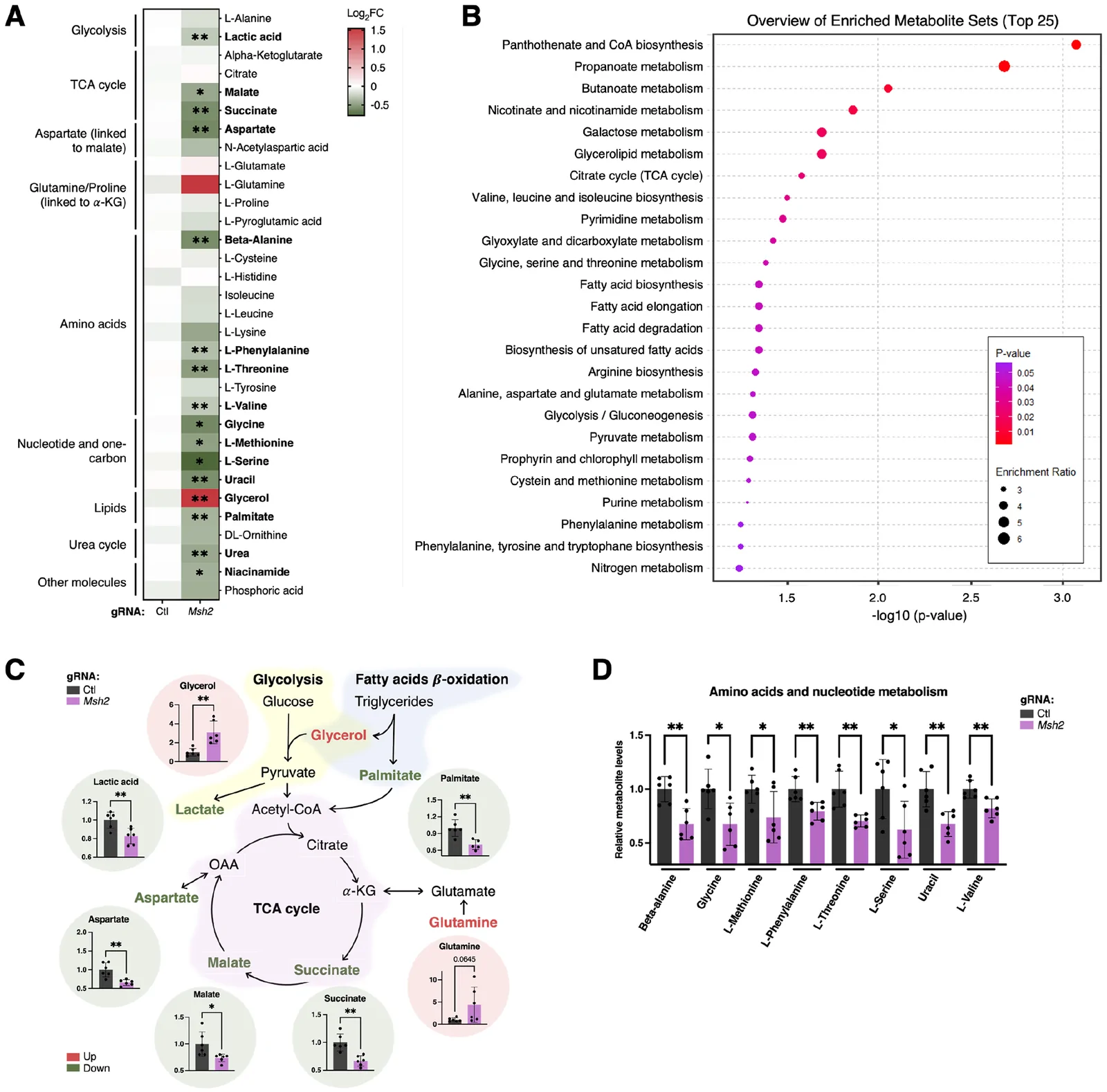
**Metabolomic analysis suggests alterations in TCA cycle and amino acid metabolism in *Msh2*-depleted organoids.** (A–D) Metabolomic analysis was performed on control (Ctl) and *Msh2*-depleted organoids (*n*=2 biological replicates, three technical replicates/biological replicate). Data were normalized to respective cell counts. (A) Heatmap showing the mean detected metabolite levels from two independent experiments performed in triplicate. Significant metabolites are in bold and their respective *P*-values are indicated. *P<0.05; **P<0.01. Color represents the log2 FC (fold change) compared to the control. (B) KEGG enriched metabolite sets significantly altered by *Msh2* loss are illustrated using MetaboAnalyst. Dot size represents the enrichment ratio, and color the *P*-value. (C) Simplified view of the TCA cycle with dysregulated metabolites is shown, where OAA stands for oxaloacetate and α-KG for α-ketoglutarate. Upregulated metabolites are in red whereas downregulated ones are in green. (D) The graph shows the fold change of metabolites linked to amino acids and nucleotide metabolism. Bar graph shows the mean±s.d. of two independent experiments performed in triplicates. *P<0.05; **P<0.01 (unpaired two-tailed Student's *t*-test).

## DISCUSSION

It is well known that stem cells exhibit a robust DNA repair capacity, and the preferred mechanism used to repair DNA damage varies according to the type of stem cell and its cellular context. As *Krt15*+ esophageal stem cells have recently been identified in mice (Giroux et al., 2017), it is important to better characterize which DNA repair pathway esophageal stem cells use to preserve their genomic integrity and maintain epithelial homeostasis. As demonstrated previously, *Msh2* is the most upregulated gene in *Krt15*+ stem cells (Giroux et al., 2017) and is associated with stemness maintenance in intestinal stem cells (Wilson et al., 1995; Tomé et al., 2013; Keysselt et al., 2017). Therefore, we hypothesized that *Msh2* would be essential in the preservation of the esophageal epithelium and the stem cell character of *Krt15*+ cells. In the predilected model to ensure stem cell maintenance (i.e. organoids), *Msh2* was depleted using the CRISPR/Cas9 method.

Upon *Msh2* depletion, esophageal organoids showed morphological alterations suggesting accumulation of basal cells. Basal cells are undifferentiated cells associated with cell proliferation. In addition, we observed an overall reduction in differentiation. Proteomic data also confirmed dysregulation of biological processes linked to epithelial cell differentiation, and of proteins directly involved in differentiation like Tgm1, Tgm3 and Sprr1a. Tgm1 and Tgm3 are transglutaminases implicated in the keratinization of the skin, and Sprr1a has previously been described as a terminal squamous cell differentiation marker (Fujimoto et al., 1997; De Heller-Milev et al., 2000; Chermnykh et al., 2020). This would be the first evidence of the possible involvement of *Msh2* in the maintenance of esophageal epithelial homeostasis. Furthermore, we observed a significant decrease in the Notch pathway target Hes1 expression, suggesting decreased Notch signaling, which would need to be further validated. This correlates with a previous study showing that inhibition of the Notch pathway with a γ-secretase inhibitor impairs esophageal differentiation (Ohashi et al., 2010; Zhang et al., 2018).

As mentioned, basal cells are highly proliferative. Thus, *Msh2*-depleted organoids displayed a noteworthy higher proliferation, as expected, without any changes in organoid diameter. This observation is consistent with other studies showing that cancer cells silenced for *Msh2* display higher cell proliferation (Nargund et al., 2022), and that cellular growth and proliferation are also affected by *Msh2* loss in intestinal stem cells (Bommi et al., 2021). As observed in human embryonic stem cells, the growth advantage conferred by *Msh2* loss was not due to changes in the cell cycle in esophageal organoids (Madden-Hennessey et al., 2022). However, increased levels of phospho-β-catenin^Ser552^ were observed in organoids depleted for *Msh2*, suggesting an activation of the Wnt/β-catenin pathway, which is crucial for stem cell proliferation in several epithelia (Van Camp et al., 2014). When phosphorylated on its serine 552, β-catenin accumulates in the nuclei and has an active transcriptional activity (He et al., 2007; Fang et al., 2007; Taurin et al., 2006). High levels of active β-catenin have also been reported in the intestinal crypts of *Msh2*^−/−^ mice (Nørgaard et al., 2020). These changes in the Wnt signaling pathway were also confirmed by proteomic analysis as an altered biological process, suggesting a possible role for the Wnt/β-catenin pathway. It has been recently proposed that Msh2 loss could regulate the Wnt/β-catenin pathway via its inhibitor DKK1 (Nørgaard et al., 2020). In brief, *Msh2* depletion in mouse esophageal organoids severely impairs the proliferation–differentiation balance, suggesting a dysregulation of tissue homeostasis and potentially cell fate decision.

As cell fate decision is typically attributed to stem cells, clonogenicity was quantified by determining the OFR. The clonogenic potential was significantly higher in *Msh2*-depleted organoids, which could imply an increase in stem cells or progenitor cells. Interestingly, the number of colonies was also significantly higher in cancer cells depleted for *Msh2* (Nargund et al., 2022). As Msh2 has been shown to play a role in cell adhesion (Nargund et al., 2022), this result might partly result from altered cell adhesion mechanisms in *Msh2*-depleted organoids, leading to a disrupted ability to grow in anchorage-independent culture. Interestingly, our proteomic data highlighted several proteins associated with cell adhesion that were differently expressed in *Msh2*-depleted organoids versus controls. For example, *Cldn4* and *Cdh1* expression, two adhesion genes, have been shown to be regulated by Msh2 in human gastric cancer (Nargund et al., 2022). Furthermore, Cdh1 is also implicated in the differentiation of keratinocytes, stemness and regeneration (Huang et al., 2021).

Because multipotency and clonogenicity in esophageal organoids are unbalanced following *Msh2* loss, homeostasis is completely disrupted under normal conditions. Interestingly, under stressed conditions, organoids depleted of *Msh2* maintained their higher clonogenic potential than control organoids even in the presence of 5-FU and cisplatin, suggesting that *Msh2* loss could reduce sensitivity to these chemotherapeutic drugs. To confirm these observations, a viability assay would need to be performed to determine IC50 values in both organoid lines. Interestingly, it has been suggested that patients with MMR-deficient gastric tumors might not benefit from fluoropyrimidine and platinum-based chemotherapy (Stolze et al., 2023), and sensitivity could vary according to the cancer stage or type (Gupta and Heinen, 2019). By contrast, *Msh2*-deficient organoids had similar sensitivity than control organoids to chemotherapeutic drugs targeting topoisomerases (i.e. etoposide and camptothecin). This contradicts a previous study that showed that colorectal cancer cells defective for *Msh2* are sensitive to those agents, supporting the notion that drug sensitivity is highly cancer type-dependent (Jacob et al., 2001). Also, the proteomic analysis herein highlighted the GO term associated with response to chemical stimulus as being enriched in *Msh2*-depleted organoids versus controls, supporting possible alteration of processes linked to resistance. Given the role of Msh2 in DNA repair, it would be interesting to compare the levels of DNA damage via γ-H2AX staining in control and *Msh2*-depleted organoids at baseline but also following treatment with chemotherapeutic drugs. Changes in clonogenicity following *Msh2* loss in basal and stressed conditions further support an increase in stem cells and/or progenitor cells. Surprisingly, the expression of most of the stem cell markers we investigated was significantly reduced in *Msh2*-depleted organoids. Stem cell markers are also downregulated in intestinal stem cells isolated from *Msh2*-depleted mice (Bommi et al., 2021). As the differentiation level is reduced and the clonogenic capacity remains high despite the reduction of stem cell markers in *Msh2*-depleted organoids, it strongly suggests an accumulation of progenitor cells that could result from the hyperproliferation of basal cells and a blockage in cell differentiation.

To further investigate the molecular mechanisms underlying the phenotypical changes observed in *Msh2*-depleted organoids, proteomic profiling was performed. This strongly highlighted alterations in several proteins associated with cell metabolism. Recent reviews are highlighting the relationship between DNA repair and metabolism (Cucchi et al., 2021; Moretton and Loizou, 2020; Sobanski et al., 2021). In our study, proteomic data showed that pathways related to DNA repair and chromatin organization are dysregulated, reinforcing the need to further investigate DNA repair capacity of *Msh2*-depleted and control organoids in the future. Proteomic profiling also revealed that many altered GO terms are related to metabolism processes. In fact, metabolomic analysis showed that 40% of the studied metabolites were significantly affected following the loss of *Msh2* in esophageal organoids, including amino acids, nucleobases, lipids and TCA cycle metabolites. These results show that a significant reprogramming of metabolism happens after the loss of Msh2. Future studies will be important to understand how, functionally, Msh2 regulates these pathways and how cells exhibiting *Msh2* depletion reprogram their metabolism. The observed increase in proliferation rates and potential differentiation of *Msh2*-depleted cells could put pressure on biosynthetic pathways, resulting in a widespread reduction in available metabolite pools.

## MATERIALS AND METHODS

### Organoid culture

Wild-type C57BL/6J mice were purchased from Charles Rivers Laboratories (#027). All animal procedures were approved by the Université de Sherbrooke Institutional Committee for the Use and Care of Animals. Esophageal cells were isolated from adult wild-type mice. The esophagus was removed, opened and rinsed in HBSS (Thermo Fisher Scientific #14175103) on ice. Tissue was incubated in a 10 U/ml dispase in HBSS solution (Thermo Fisher Scientific #CB-40235). The epithelium was then peeled from the rest of the tissue and digested with trypsin (Wisent #325-043-CL) to retrieve cells. A single-cell suspension was filtered and then cultured as organoids in Matrigel^®^ (Corning™ #354230) with mouse organoid esophageal medium [advanced DMEM-F12 (Gibco™ #12634010) supplemented with 1× GlutaMAX™ (Gibco™ #35050061), 1X HEPES (Wisent #330-050-EL), 1× penicillin-streptomycin (Wisent #450-201-EL), 1X N-2 Supplement (Thermo Fisher Scientific #17502048), 1X B-27™ Supplement (Gibco™ #17504044), 0.1 mM N-acetylcysteine (Sigma-Aldrich #A7250-5G), 0.05 µg/ml mouse recombinant EGF (PreproTech #315-09), 3% Noggin and R-Spondin conditioned medium and 10 µM Y27632 (AdooQ Bioscience #A11001-50)]. Medium was changed every other day. Organoids were passaged every 7 days through enzymatic digestion (Giroux et al., 2017).

### Establishment of *Msh2*-depleted organoids

Plasmids containing the CRISPR/Cas9 sequence and guide RNAs (gRNAs) targeting the mouse *Msh2* gene were generated as described previously (Sanjana et al., 2014; Shalem et al., 2014). Briefly, lentiCRISPRv2 plasmid (Addgene #52961) was used to insert oligonucleotides of *Msh2* gRNA #1 (5′-CCTTAATAAATGCAGCCCGG-3′ and 5′-GGAATTATTTACGTCGGGCC-3′). For control, an empty vector was used. Lentiviruses were produced in HEK293T cells using the ViraPower™ Lentiviral Expression System (Invitrogen #25-0501) and Lipofectamine 3000 kit (Thermo Fisher Scientific #L3000015) according to the manufacturer's recommendations. Supernatant was harvested 48 h post-transfection and filtered. Viruses were concentrated using the Lenti-X™ concentrator (Takara Bio #631231) according to the manufacturer's recommendations. For the infection**,** cells dissociated from wild-type organoids were mixed with polybrene (8 µg/ml) and concentrated lentiviruses, and then transferred to a low-attachment 96-well plate for spinfection [800 ***g*** for 1 h at room temperature (RT)]. Cells were then resuspended in Matrigel^®^ and grown in organoid medium for a week. At day 7, organoids were passaged 1:1, and selection was started by adding 2 µg/ml puromycin to the medium. Selection media was changed every day. CRISPR/Cas9-edited organoid populations were then used for both conditions.

### Area measurements

Area of organoids was measured with Fiji (Image J version 2.0.0-rc-69/1.52p).

### Viability

At 5 days post-seeding, organoids were brought into a single-cell suspension before being treated with VivaFix™ Cell Viability Assay (Bio-Rad #135-1115) according to the manufacturer's recommendations. Cells were analyzed with BD LSR Fortessa™ flow cytometer and using FlowJo 10.8.1 software.

### Organoid formation assay

300 cells were seeded in quadruplicate and grown as organoids in a 48-well plate. On day six, organoids were manually counted for each population. The organoid formation rate (OFR) refers to the percentage of seeded cells that successfully grow as organoids. For OFR assay under stress condition, organoids were incubated from day 1 (24 h post-seeding) with chemotherapeutic agents: 10 µM etoposide [Selleck Chemicals # S1225-100MG(CA)], 30 µM cisplatin (Cayman Chemicals #13119), 50 nM camptothecin (Cayman Chemicals #11694), 10 µM 5-fluorouracil (5-FU; Cayman Chemicals #14416) or vehicle for 72 h.

### WST-1 assay

1000 cells were seeded in quadruplicate and grown as organoids in 5 µl Matrigel^®^ per well in 96-well plates. At day 1, 3, 5 and 7 post-seeding, cell proliferation was measured with WST-1 reagent (Roche #5015944001) according to the manufacturer's recommendations. Absorbance was measured at 440 nm with a spectrophotometer.

### Flow cytometry

At 7 days post-seeding, the organoids were harvested using dispase and brought to a single-cell suspension. Cell pellets were washed in FACS buffer (1% BSA in HBSS) before being incubated with FITC anti-mouse CD73 (BioLegend #127219, RRID:AB_2716075) for 30 min in the dark at RT. After being washed in FACS buffer once, cells were analyzed with a BD LSR Fortessa™ flow cytometer and using FlowJo™ software version 10.8.1 (https://www.flowjo.com).

### Western blotting

Organoids were recovered from Matrigel^®^ with dispase and then lysed in RIPA buffer supplemented with protease inhibitor cocktail (Thermo Fisher Scientific), 10 mM sodium fluoride, 40 mM β-glycerophosphate and 200 µM orthovanadate. Protein extracts were separated on SDS-PAGE and transferred to PVDF membranes. Following blocking, membranes were incubated with primary antibodies overnight at 4°C. Primary antibodies were used against the following proteins at the following dilutions: Msh2 (1:1000, Cell Signaling Technology #2017, RRID:AB_2235387), Msh6 (1:1000, Abcam #ab92471, RRID:AB_2144959), Hes1 (1:1000, Cell Signaling Technology #11988, RRID:AB_2728766), phospho-histone H3^S10^ (1:1000, Cell Signaling Technology #9701, RRID:AB_331535), phospho-β-catenin^Ser552^ (1:500, Cell Signaling Technology #9566, RRID:AB_1031116), cleaved caspase-3 (1:1000, Cell Signaling Technology #9664, RRID:AB_2070042), p21 Waf1/Cip1 (1:1000, Cell Signaling Technology #64061), p27 Kip1 (1:1000, Cell Signaling Technology #3698, RRID:AB_2077832) and actin, clone C4 (1:5000, Millipore #MAB1501, RRID:AB_2223041). Membranes were then incubated with HRP-labeled secondary antibodies: anti-rabbit IgG (1:5000, Cell Signaling Technologies #7074, RRID:AB_2099233) and anti-mouse IgG (1:5000, Cell Signaling Technologies #7076, RRID:AB_330924) for 1 h at RT and proteins of interest were visualized using Clarity Max™ ECL (Bio-Rad) or Immobilon^®^ Forte (Millipore) substrates. Full blots are available in Fig. S3.

### Immunofluorescence

Organoids from both populations were harvested under the same conditions at the same time. Organoids were fixed in zinc-formalin (Richard-Allan Scientific) overnight at 4°C and then resuspended in melted embedding solution (2% agarose and 2.5% gelatin). Organoids were paraffin-embedded and 5 µm sections were cut. After sections were deparaffinized, antigen retrieval was performed in pH 6.0 citric acid buffer. Sections were then blocked and incubated overnight at 4°C in primary antibodies against: Msh2 (1:200, Cell Signaling Technology #2017, RRID:AB_2235387), Ki67 (1:200, Thermo Fisher Scientific #MA5-14520, RRID:AB_10979488) and Lamin B (1:300, Santa Cruz Biotechnology #sc-6217, RRID:AB_648158). Involucrin primary antibody (1:600, Thermo Fisher Scientific #PA5-104453, RRID:AB_2853754) was incubated for 1 h at RT. The next day, slides were incubated with secondary antibodies: anti-rabbit IgG DyLight^®^ 549 (1:400, Vector Laboratories #DI-1549, RRID:AB_2336407) and anti-goat IgG DyLight^®^ 488 (1:400, Vector Laboratories #DI-3088, RRID:AB_2336400) for 90 min at RT and mounted using Vectashield with DAPI (Vector Labs #H-2000). Sections were visualized using a Zeiss Axioscope 5 fluorescence microscope and a Zeiss LSM880 confocal multiphoton laser scanning microscope, both using Zen blue acquisition software. Optical sections were analyzed using Fiji/Image J version 2.0.0-rc-69/1.52p (Schindelin et al., 2012). CellProfiler version 3.1.5 (McQuin et al., 2018) was used to determine the nuclear circularity, also called form factor, by identifying the nuclei marked with lamin B. A form factor of 1 represents a perfectly circular nuclear shape.

### Organoid morphology and diameter

Hematoxylin and eosin (H&E) coloration was performed on organoid sections to investigate morphology. Images were obtained with a digital slide scanner Hamamatsu Nanozoomer 2.0-RS. Morphology was assessed using a classification system that was developed according to pre-selected images (Fig. S1A). We can then classify organoids as well-differentiated, intermediate and poorly differentiated. Organoids were counted by two independent individuals. Diameter was measured with the NDP.view2 software from Hamamatsu.

### RNA preparation and qPCR

Total RNA was extracted from harvested organoids with the RNeasy Plus Mini kit (Qiagen #74134) and reverse transcribed with SuperScript™ III Reverse Transcriptase (Invitrogen #18080044) according to the manufacturer's recommendations. qPCR was performed using FastStart Essential DNA Green Master (Roche # 06402712001) in a LightCycler^®^ 480 System (Roche). The following mouse primers were used and *Gapdh* was used as an internal control (Table S2).

### Mass spectrometry

#### Digestion of protein total extract for data-independent acquisition mass spectrometry

Three independent biological replicates per group were used in data-independent acquisition mass spectrometry (DIA-MS). Protein extraction of control and *Msh2*-depleted organoids was performed using a solution of 8 M urea, 1 M NH_4_HCO_3_ and 20 mM HEPES pH 8.0. On ice, samples were sonicated for 60 s by 5 s intervals at 20% amplitude (FB120 Sonic Dismembrator, Thermo Fisher Scientific) and then centrifuged at 16,000 ***g*** for 10 min at 4°C. Protein concentration was determined by BCA assay. 30 μg of proteins were reduced by adding dithiothreitol to a final concentration of 5 mM. Samples were incubated for 2 min at 95°C, followed by 30 min at RT. Protein alkylation was completed by the addition of chloroacetamide to a final concentration of 7.5 mM for 20 min in the dark at RT. Urea concentration was diluted to a final concentration of 2 M by adding 50 mM ammonium bicarbonate. For protein digestion, 1 μg of MS-grade trypsin was added to each sample and incubated overnight at 30°C with agitation.

#### Purification and desalting of peptides using C18 columns for DIA-MS

Digestion was stopped by adding trifluoroacetic acid to a final concentration of 0.2%. According to the manufacturer's recommendations, digested peptides were purified with C18 tips (Thermo Fisher Scientific Pierce). Samples were dried at 60°C for 3 h using a centrifugal evaporator, then resuspended in 1% formic acid (FA).

#### Fractionation of digested samples for the DIA library

Samples were combined into one to create the library, which was fractionated using Pierce high pH reversed-phase peptide fractionation kit (Thermo Fisher Scientific #84868) according to the manufacturer's recommendations. Peptides were eluted in eight fractions and dried using a centrifugal evaporator at 60°C for approximately 3 h. Samples were then resuspended in 1% FA. The DIA library was then analyzed by data-dependent acquisition (DDA).

#### DDA LC-MS analysis for the DIA library

From each fraction, 250 ng of peptides were injected into a high-performance liquid chromatography (HPLC) machine (nanoElute, Bruker Daltonics) and loaded onto a trap column with a constant flow of 4 µl/min (Acclaim PepMap100 C18 column, 0.3 mm id×5 mm, Dionex Corporation). Elution was done onto an analytical C18 column (1.9 µm bead size, 75 µm×25 cm, PepSep) over a 2-h gradient of acetonitrile (5–37%) in 0.1% FA at 400 nl/min while being injected into a TimsTOF Pro ion mobility mass spectrometer equipped with a Captive Spray nano electrospray source (Bruker Daltonics). Data was acquired using data-dependent auto-MS/MS with a 100–1700 *m*/*z* mass range, with PASEF enabled with PASEF scans set at a number of 10 (1.17 s duty cycle) and a dynamic exclusion of 0.4 min, *m*/*z*-dependent isolation window and collision energy of 42.0 eV. The intensity threshold was set at 2500, and the target intensity was set at 20,000. Mass spectrometry experiments were performed at the proteomic and mass spectrometry platform at Université de Sherbrooke.

#### Protein identification for DIA library – TIMS

Using MaxQuant software version 2.0.3.0 (Cox and Mann, 2008; Tyanova et al., 2016) and the Uniprot mouse proteome database (02/2022, 55,286 entries), the raw files (eight for each organoid line) were analyzed to obtain the DIA library. The following settings were used for the MaxQuant analysis [with group-specific thermal ionization mass spectrometry (TIMS)-DDA type parameters]: 1 miscleavage was authorized; cysteine carbamidomethylation was a fixed modification; trypsin was the enzyme (K/R was not before P); methionine oxidation and protein N-terminal modification were included in the analysis; precursor and fragment ions had a mass tolerance of 20 ppm; ‘peptide-spectrum matching (PSM) false discovery rate (FDR)’, ‘Protein FDR’, and ‘Site decoy fraction’ identification values were set to 0.05; 1 was the minimum peptide count; ‘Match between runs’ and ‘Second peptides’ were permitted; a transfer q value of 0.3 was used to run MaxQuant. From this DDA analysis, the ‘peptides.txt’, ‘evidence.txt’ and ‘msms.txt’ files were used for DIA analysis.

#### DIA LC-MS analysis

Data was acquired with diaPASEF mode and using the parameters described for DDA analysis. One mobility window with 27 mass steps (*m*/*z* between 114 and 1414 with a mass width of 50 Da) per cycle (1.27 s duty cycle) was used for each single TIMS (100 ms) in diaPASEF mode, which covers in the *m*/*z*-ion mobility plane the diagonal scan line for charged peptides +2 and +3.

#### Protein identification for DIA-MS

MaxQuant (version 2.0.3.0) and the UniProt mouse proteome database (02/2022, 55,286 entries) were used to analyze the raw files. Settings were as described above, except for the following: MaxQuant and TIMS MaxDIA were selected; 3 files (“peptides.txt,” “evidence.txt,” and “msms.txt”) from the DIA library were used.

#### Data analysis for DIA-MS

Single peptides and proteins positive for ‘Reverse’, ‘Only identified by site’ or ‘Potential contaminant’ categories were excluded. Proteomic data were then analyzed using Advaita's Bio iPathwayGuide (Draghici et al., 2007; Donato et al., 2013) with a minimum peptide count of 2, with a threshold of 1.5 for log2 fold-change (calculated with lfq-intensity) and with 0.05 for *P*-value. As no correction was applied to identify gene ontology (GO) terms and biological processes, a minimum of 20 differentially expressed genes that significantly changed was applied to make the analysis more stringent. Graphs were obtained using R software version 4.2.2. Network was obtained using the Cytoscape software version 3.9.1 (Shannon et al., 2003). Proteomics data have been deposited to the ProteomeXchange Consortium via the PRIDE (Perez-Riverol et al., 2022) partner repository with the dataset identifier PXD040810.

### Metabolomic analysis

Organoids were prepared for metabolomics analysis as described previously (Frégeau-Proulx et al., 2022; Lacouture et al., 2021). Briefly, four Matrigel drops containing 20,000 cells each were plated in six-well plates in triplicate for both populations, and 1.5 ml of medium was added to each well. As a control, two Matrigel drops without cells were also plated in medium. Medium was changed 4 days post-seeding only. At 7 days post-seeding, plates were put on ice, medium was removed from the six-well plate, and wells were rinsed using cold 0.9% NaCl. Organoids were then harvested in dry ice-cold 80% methanol (HPLC-grade) using a cell scraper and stored at −80°C until analysis. In parallel, two Matrigel drops containing 20,000 cells were plated in 48-well plates for each population for normalization. At 7 days post-seeding, organoids were harvested using dispase and brought to a single-cell suspension in the same tube for precise cell counting to normalize data. Two independent biological replicates per group were used and three technical replicates for each biological replicate.

On the day of analysis, samples were derivatized using a two-step protocol: (1) methoxyamination with the method described by Fiehn (2016) and (2) silylation with *N*-methyl-*N-*tert-butyldimethylsilyltrifluoroacetamide/tert-butyldimethylchlorosilane using the modified method from Patel and collaborators (Patel et al., 2017). Samples were then analyzed with gas chromatography (GC)-MS using an Agilent 8890 GC equipped with a DB5-MS+DG capillary column connected to an Agilent 5977B MS operating under electron impact (EI) ionization at 70 eV (Agilent Technologies, Santa Clara, CA, USA), as previously described (Frégeau-Proulx et al., 2022; Lacouture et al., 2021). The Agilent MassHunter Workstation Software was then used for analysis and, after deconvolution, the NIST/EPA/NIH Mass Spectral Library (NIST 2017, Gaithersburg, MD, USA) was used to identify target metabolites. Finally, relative metabolite levels were normalized with the internal standard Myristic acid-d27 (CDN Isotopes, Canada) and with the cell number. Metabolomics data were studied using MetaboAnalyst 5.0 (Pang et al., 2021). For the heatmap, data were transformed into log2 for visualization. Metabolomics analyses were performed at the metabolomic platform of Université de Laval.

### Statistics

Statistical analyses were calculated with GraphPad Prism 9 Version 9.3.1. Tests were corrected for variance, multiple comparisons and normality. *P*-values were determined using the unpaired two-tailed Student's *t*-test where *P<0.05; **P<0.01; ***P<0.001 and ****P<0.0001.

## Supplementary Material



10.1242/joces.264470_sup1 Supplementary information

Table S1. List of differentially expressed proteins in Msh2-depleted organoids
