# Supplementary material for: Msh2 depletion impairs cell differentiation, proliferation and metabolism in esophageal organoids
Source: J Cell Sci. 2026 Oct 19;139(19):jcs264470. doi: 10.1242/jcs.264470 (PMC13723390; doi:10.1242/jcs.264470)
Supplement: Supplementary file 1 [file joces-139-264470-s1.pdf]

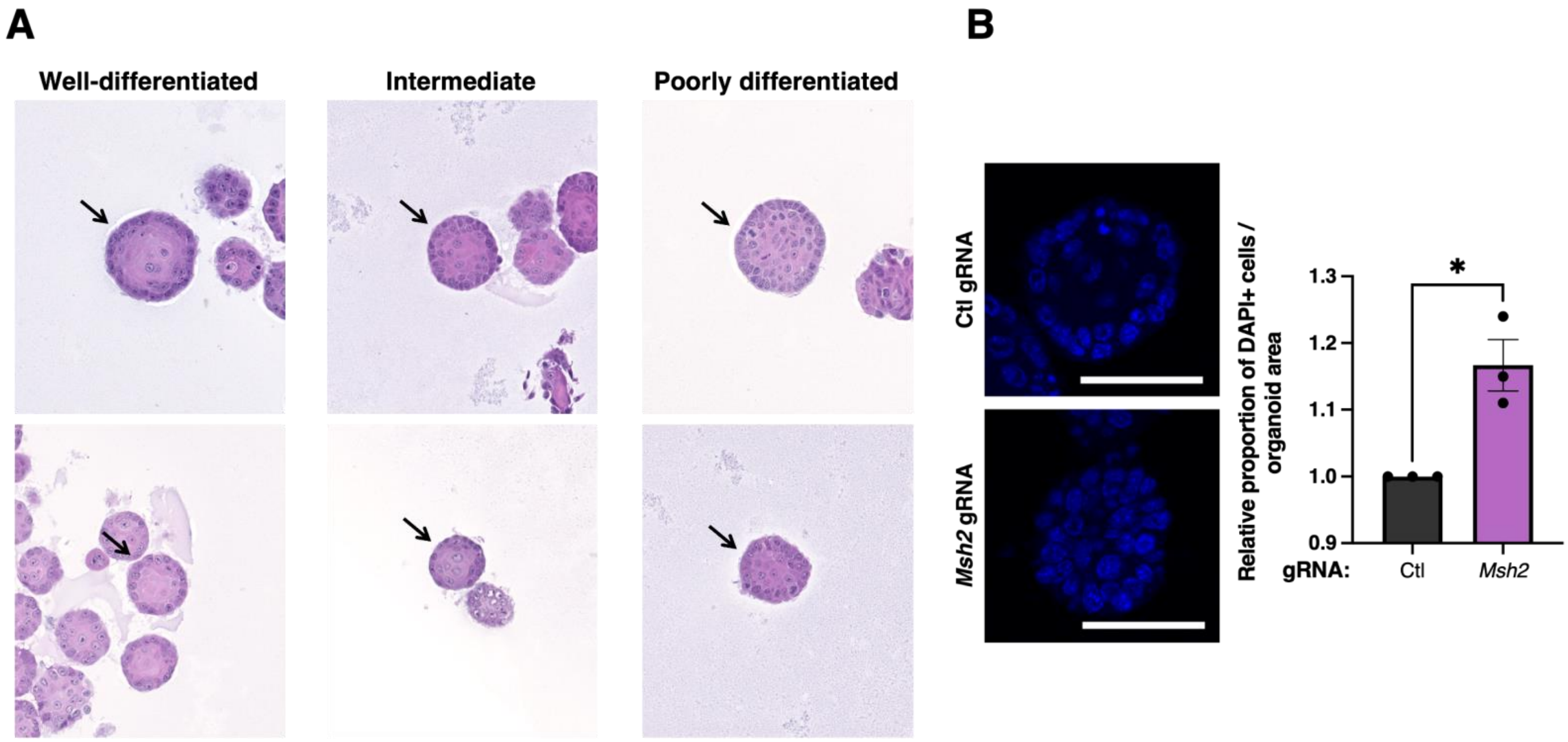


**Fig. S1. Organoid morphology is altered by *Msh2* loss.**

**A.** Organoids were categorized according to 3 different levels of differentiation. Representative images of each state are shown. The well-differentiated organoids possess one well defined layer of basal cells and a pink keratinized center while the poorly differentiated organoids have basal cells in the center and absence of keratinization. The intermediate phenotype is characterized by a disorganized basal cell layer and an inferior keratinization level. **B.** Immunofluorescence of DAPI+ cells of organoids of similar size. Graph shows the relative proportion of DAPI+ cells relative to the organoid area (n=3, count of 17-34 organoids/condition). Scale bar = 50 µm. p-values were determined by unpaired two-tailed Student's t-test where * p < 0,05.

Journal of Cell Science • Supplementary information

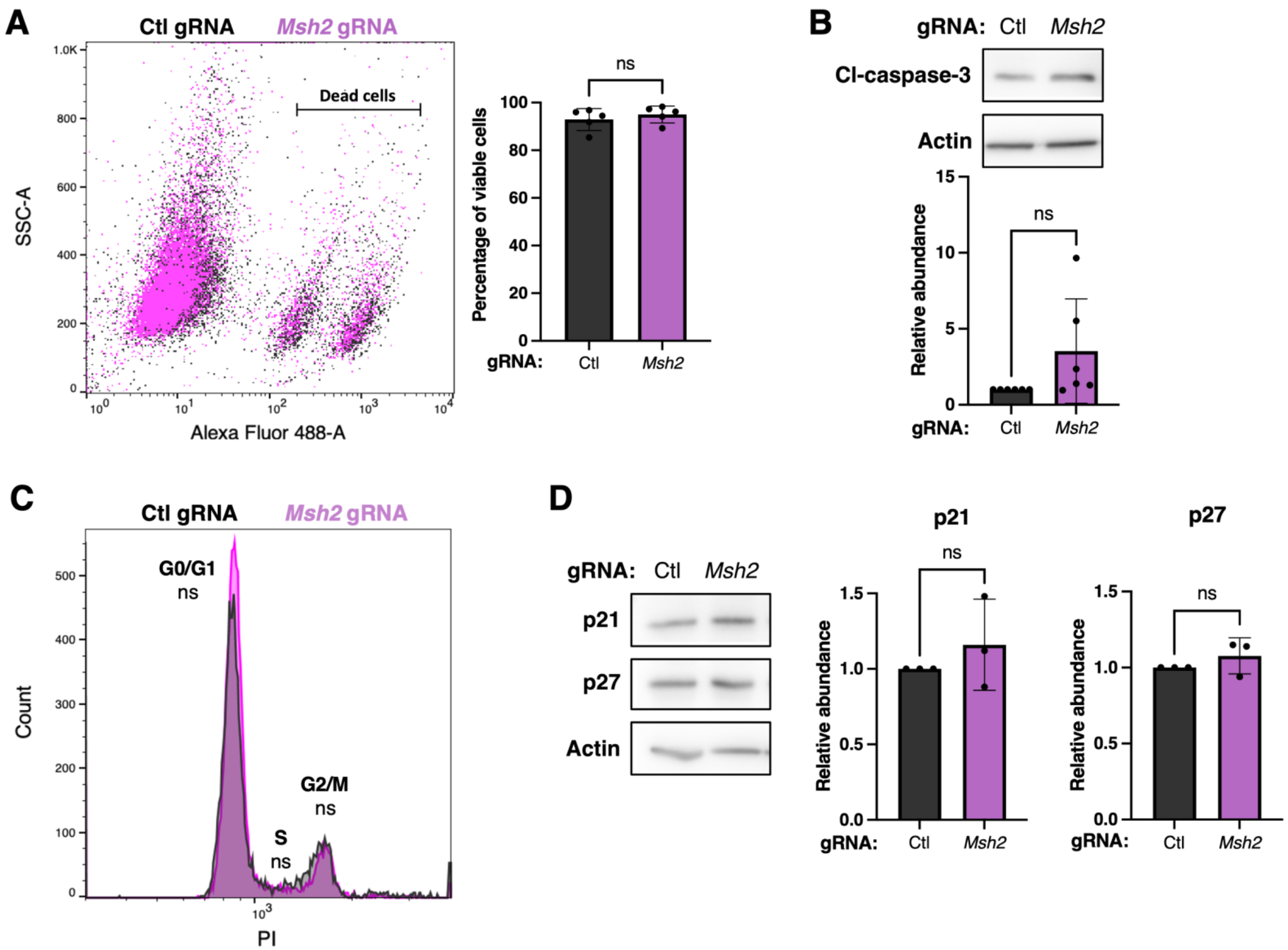


**Fig. S2. *Msh2* depletion does not affect the viability or cell cycle progression. A-D.** *Msh2* was depleted in mouse esophageal organoids using the CRISPR/Cas9 approach. **A.** Viability was assessed using VivaFix™ dye. Graph shows the percentage of viable cells. Bar graph shows mean ± SD (n=4, count of 10,000 events). **B.** Western blot for cleaved caspase-3 was performed using actin as a loading control (n=6). **C.** Propidium iodide was used to assess cell cycle phases by flow cytometry (n=3). **D.** Western blot for the cell cycle inhibitors p21 and p27 was done using actin as a loading control (n=3). p-values were determined by unpaired two-tailed Student's t-test where ns = non-significant; * p < 0,05.

Journal of Cell Science • Supplementary information

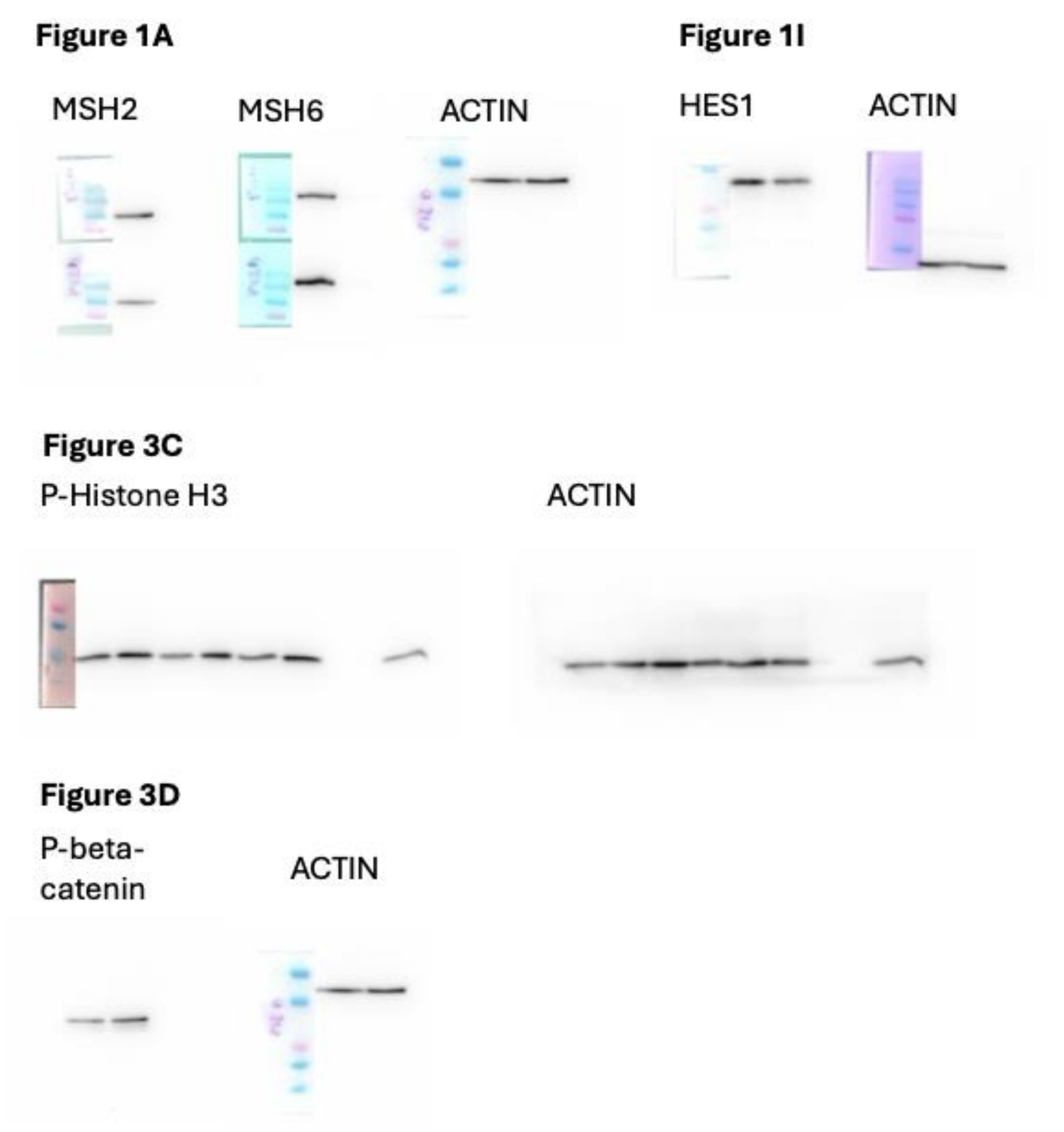


**Fig. S3. Blot transparency**

Journal of Cell Science • Supplementary information

## Table S1. List of differentially expressed proteins in *Msh2*-depleted organoids

Available for download at
https://journals.biologists.com/jcs/article-lookup/doi/10.1242/jcs.264470#supplementary-data

## Table S2. Primers used for qPCR (mouse genes)

| Gene | Forward primer | Reverse primer |
|---|---|---|
| *Krt13* | GACTGGCATCTGAAACAGAGCC | TTGTCCGTGGTGGCTTCCAGA |
| *Notch3* | GGTAGTCACTGTGAACACGAGG | CAACTGTCACCAGCATAGCCAG |
| *Wnt5a* | GGAACGAATCCACGCTAAGGGT | AGCACGTCTTGAGGCTACAGGA |
| *CD73* | GACACTCCAACACCTTTCTCTAC | CATCATCTGCGGTGACTATGAA |
| *Lrig1* | GAACACCTGAACCTTGGAGAG | CTGCAGCATCCTACCCATTAG |
| *Prom2* | CACCATCCATGAACCTATCCTATC | CAGGTGCTTATCCAGGTCATAC |
| *Smoc2* | TCGGCAGAACAAGACCAATAA | CTAGCACACACCAGCAGTATC |
| *Bmi1* | CCAGACCACTCCTGAACATAAG | CTTCTCCTCGGTCTTCATTGG |
| *Gapdh* | GGGTGTGAACCACGAGAAATA | AGTGATGGCATGGACTGTG |

Journal of Cell Science • Supplementary information
