# Supplementary material for: Msh2 depletion impairs cell differentiation, proliferation and metabolism in esophageal organoids
Source: J Cell Sci. 2026 Oct 19;139(19):jcs264470. doi: 10.1242/jcs.264470 (PMC13723390; doi:10.1242/jcs.264470)
Supplement: Supplementary file 2 [file joces-139-264470_review_history.pdf]

Journal of Cell Science

# Msh2 depletion impairs cell differentiation, proliferation and metabolism in esophageal organoids

Marjorie Rolland, Dominique Jean, Maude Hamilton, Molly Sedeuil, Alexis Gonneaud, Line Berthiaume, Étienne Audet-Walsh and Véronique Giroux
DOI: 10.1242/jcs.264470

**Editor**: Aryeh Warmflash

**Review timeline**

| | |
|---|---|
| Original submission: | 23 September 2025 |
| Editorial decision: | 17 November 2025 |
| First revision received: | 3 March 2026 |
| Accepted: | 13 March 2026 |


---

**Original submission**

First decision letter

MS ID#: jcs.264470

MS TITLE: Msh2 depletion impairs cell differentiation, proloferation and metabolism in esophageal organoids

AUTHORS: Marjorie Rolland; Dominique Jean; Maude Hamilton; Molly Sedeuil; Alexis Gonneaud; Line Berthiaume; Étienne Audet-Walsh; Véronique Giroux
ARTICLE TYPE: Research Article

Dear Dr Giroux,

We have now reached a decision on the above manuscript.

As you will see, the reviewers gave favourable reports but raised some critical points that will require amendments to your manuscript. I hope that you will be able to carry these out because I would like to be able to accept your paper, depending on further comments from reviewers.

You will note that both reviewers raise technical points which should be addressed. Reviewer 1 also raises concerns that the data are too preliminary and requests additional mechanistic insight while Reviewer 2 raises a similar concern but notes that this could be addressed in the discussion without requiring additional experiments. I agree with reviewer 2 and do not believe that additional experiments should not be required for this purpose.

Reviewer 1

SUMMARY OF THE ADVANCE MADE IN THIS PAPER AND ITS POTENTIAL SIGNIFICANCE TO THE FIELD

This study investigates how loss of the DNA mismatch repair gene Msh2 affects epithelial differentiation, proliferation, and metabolism in mouse esophageal organoids. The authors used CRISPR/Cas9-mediated knockout in organoids and combined morphological, transcriptional, proteomic, and metabolomic analyses. They report that Msh2-deficient organoids show reduced differentiation, increased proliferation and clonogenicity, and altered metabolic profiles.

© 2026. Published by The Company of Biologists under the terms of the Creative Commons Attribution License (https://creativecommons.org/licenses/by/4.0/).

The topic is relevant to epithelial stem cell biology and DNA repair, and the integration of multi-omics data within an organoid model is conceptually sound. However, the study remains largely descriptive, lacks mechanistic depth, and omits key methodological details required for reproducibility. In its current form, the work appears too preliminary for publication in Journal of Cell Science.

SUGGESTIONS TO AUTHORS

Major comments [Please request additional experiments only if they are essential for supporting the conclusions; authors should be encouraged to highlight any claims that are preliminary or speculative, or to discuss any pitfalls or alternative interpretations in a 'Limitations' section]

- Gene editing and validation: Include indel validation (Sanger/ICE or amplicon sequencing) at sgRNA-targeted regions to confirm on-target editing. Provide quantitative data confirming Msh2 loss at both mRNA and protein levels.
Use multiple independent Msh2 gRNA clones or complementary knockdown approaches to strengthen reproducibility and generalizability. The current design, based on a single clone, limits interpretability.

- Statistical rigor in omics analyses: For both proteomic and metabolomic analyses, indicate the number of biological replicates per group and clarify whether technical replicates were performed. This information is missing from the Methods and Figure legends but is essential for evaluating statistical robustness. Explicitly report replicate numbers, missing-value handling, normalization approach, and multiple-testing correction (e.g., FDR). Without these, the omics findings cannot be assessed for reliability.

- Drug-response data: The claims regarding 5-FU and cisplatin response differences are under-supported. Validate these observations with at least one independent viability or apoptosis assay, and report $IC_{50}$ values or relative survival with standard deviations.

- Mechanistic insight and validation: 1) The manuscript proposes that Msh2 loss alters differentiation and metabolism but provides no mechanistic explanation. Key pathways are only inferred from proteomic signatures and not experimentally validated. Include additional functional assays or molecular readouts to demonstrate causal links—such as examining HES1, AXIN2, γH2AX, or other pathway markers by qPCR or Western blot—and confirm that altered signaling drives the observed phenotypes. 2) Incorporate a rescue experiment or a second independent Msh2 knockout line. 3) Functionally validate a key pathway alteration suggested by the omics data. 4) Confirm selected proteomic/metabolomic hits by independent assays.

Minor comments

- Add molecular weight (kDa) to all Western-blot figures.

- Add quantitative results for Figure 1D

Reviewer 2

SUMMARY OF THE ADVANCE MADE IN THIS PAPER AND ITS POTENTIAL SIGNIFICANCE TO THE FIELD

This manuscript investigates the effects of Msh2 loss in murine esophageal organoids. The authors used Crispr/Cas9 gene editing to delete Msh2 in esophageal organoids and found that Msh2 loss impacted cell differentiation, proliferation and metabolism. The authors report that loss of Msh2 resulted in decreased cell differentiation, and increased proliferation and clonogenicity, this was accompanied by alterations in metabolic processes. Overall, the manuscript is well written and organized, the experiments are logical, well planned and the figures/data support the authors' major points.

Major comments:

© 2026. Published by The Company of Biologists under the terms of the Creative Commons Attribution License (https://creativecommons.org/licenses/by/4.0/). 

1. Since Msh2 has a role in DNA repair, do Msh2 depleted organoids have increased DNA damage or exhibit DNA damage at baseline or after prolonged culturing or other non-chemotherapeutic treatment? Staining for yH2AX or other proteins in DNA damage/repair could help support this argument in organoids. I do not view this as an essential experiment, it could be addressed in the discussion.

Minor comments:

1. Some editing of the manuscript is necessary. There are some typos, including in the title where proliferation is mis-spelled. The last paragraph of the discussion has several sentences that have four or five words repeated consecutively. In the supplemental methods under viability, analyzed is repeated.
2. It is unclear from the figure legends what n represents (in most figure legends), is it number of organoids, number of mice the organoids were derived from and then gene edited, number of replicates, etc? What the graphs/data represent should be clearly stated.
3. Including the percentage of conditioned media for R-spondin and noggin used would help others reproduce organoid results in the future.

---

**First revision**

<u>Author response to reviewers' comments</u>

We the editor for the opportunity to resubmit our work to the *Journal of Cell Science*. We are pleased to provide a revised version of our manuscript entitled "*Msh2* depletion impairs cell differentiation, proliferation and metabolism in esophageal organoids" (manuscript # jcs.264470).

We are also grateful to both reviewers for their thoughtful and constructive comments. Although no additional experiments were performed, as recommended by the editor, we have addressed all concerns either in the Discussion or Methods sections. Below, we provide a detailed point-by-point response.

**<u>EDITOR</u>**

***Comment 1.1***: *"As you will see, the reviewers gave favourable reports but raised some critical points that will require amendments to your manuscript. I hope that you will be able to carry these out because I would like to be able to accept your paper, depending on further comments from reviewers. You will note that both reviewers raise technical points which should be addressed. Reviewer 1 also raises concerns that the data are too preliminary and requests additional mechanistic insight while Reviewer 2 raises a similar concern but notes that this could be addressed in the discussion without requiring additional experiments. I agree with reviewer 2 and do not believe that additional experiments should not be required for this purpose."*

**Reply 1.1**: As suggested, we have addressed the reviewers' concerns into the Discussion or Methods sections. Details are provided below.

**<u>REVIEWER 1</u>**

***Comment 2.1***: *"Gene editing and validation: Include indel validation (Sanger/ICE or amplicon sequencing) at sgRNA-targeted regions to confirm on-target editing. Provide quantitative data confirming Msh2 loss at both mRNA and protein levels.*
*Use multiple independent Msh2 gRNA clones or complementary knockdown approaches to strengthen reproducibility and generalizability. The current design, based on a single clone, limits interpretability"*

© 2026. Published by The Company of Biologists under the terms of the Creative Commons Attribution License (https://creativecommons.org/licenses/by/4.0/).

**Reply 2.1**: Loss of *Msh2* was validated at the protein level by Western blotting (Figure 1A) and immunofluorescence (Figure 1B). While we agree that including an additional *Msh2* gRNA would have strengthened the study, generating independent clones in an organoid system is technically challenging. To reduce the risk of clone-specific effects, we opted to perform experiments in bulk populations. Three gRNAs were initially tested, and the only one that fully eliminated Msh2 protein expression was used for the present study. As recommended by the editor, no additional experiments were performed, but we have clarified these methodological details in the revised Methods section.

***Comment 2.2***: *"Statistical rigor in omics analyses: For both proteomic and metabolomic analyses, indicate the number of biological replicates per group and clarify whether technical replicates were performed. This information is missing from the Methods and Figure legends but is essential for evaluating statistical robustness. Explicitly report replicate numbers, missing-value handling, normalization approach, and multiple-testing correction (e.g., FDR). Without these, the omics findings cannot be assessed for reliability."*

**Reply 2.2**: Proteomic analyses were performed using three biological replicates per group, and the metabolomic analyses used two biological replicates per group with three technical replicates for each. These details have been added to the Methods and figure legends.

***Comment 2.3***: *"Drug-response data: The claims regarding 5-FU and cisplatin response differences are under-supported. Validate these observations with at least one independent viability or apoptosis assay, and report $IC_{50}$ values or relative survival with standard deviations."*

**Reply 2.3**: We thank the reviewer for this suggestion. Although we did not perform additional experiments, we have refined our claims accordingly in the Abstract, Methods, Results, and Discussion sections.

***Comment 2.4***: *"Mechanistic insight and validation: 1) The manuscript proposes that Msh2 loss alters differentiation and metabolism but provides no mechanistic explanation. Key pathways are only inferred from proteomic signatures and not experimentally validated. Include additional functional assays or molecular readouts to demonstrate causal links—such as examining HES1, AXIN2, γH2AX, or other pathway markers by qPCR or Western blot—and confirm that altered signaling drives the observed phenotypes. 2) Incorporate a rescue experiment or a second independent Msh2 knockout line. 3) Functionally validate a key pathway alteration suggested by the omics data. 4) Confirm selected proteomic/metabolomic hits by independent assays"*

**Reply 2.4**: We appreciate these valuable suggestions for future work. However, these experiments are beyond the scope of the present manuscript.

***Comment 2.5***: *"Minor comment: Add molecular weight (kDa) to all Western-blot figures."*

**Reply 2.5**: Full blots with ladder are now provided included in Supplemental Figure S3.

***Comment 2.6***: *"Minor comment: Add quantitative results for Figure 1D".*

**Reply 2.6**: Quantification of organoid diameter and differentiation status is included in Figure 1E and 1F, respectively. We have refined the text to avoid ambiguity.

<u>**REVIEWER 2**</u>

***Comment 3.1***: *"Since Msh2 has a role in DNA repair, do Msh2 depleted organoids have increased DNA damage or exhibit DNA damage at baseline or after prolonged culturing or other non-chemotherapeutic treatment? Staining for γH2AX or other proteins in DNA damage/repair could*

© 2026. Published by The Company of Biologists under the terms of the Creative Commons Attribution License (https://creativecommons.org/licenses/by/4.0/).

*help support this argument in organoids. I do not view this as an essential experiment, it could be addressed in the discussion."*

**Reply 3.1**: We agree that comparing baseline DNA damage and repair capacity between *Msh2*-depleted and control organoids would be informative. This has now been added the Discussion.

***Comment 3.2****: "Some editing of the manuscript is necessary. There are some typos, including in the title where proliferation is mis-spelled. The last paragraph of the discussion has several sentences that have four or five words repeated consecutively. In the supplemental methods under viability, analyzed is repeated."*

**Reply 3.2:** We apologize for these errors. All typos and repeated words have been corrected.

***Comment 3.3****: "It is unclear from the figure legends what n represents (in most figure legends), is it number of organoids, number of mice the organoids were derived from and then gene edited, number of replicates, etc? What the graphs/data represent should be clearly stated."*

**Reply 3.3**: Clarifications for all figure legends have been added. Additional details regarding replicate numbers and experimental design appear in the Methods. Briefly, most experiments were performed using different biological replicates of a single gene-edited organoid line.

***Comment 3.4****: "Including the percentage of conditioned media for R-spondin and noggin used would help others reproduce organoid results in the future."*

**Reply 3.4**: The percentage is now included in the Methods section.

---

Second decision letter

MS ID#: jcs.264470R1

MS Title: Msh2 depletion impairs cell differentiation, proliferation and metabolism in esophageal organoids

Authors: Marjorie Rolland; Dominique Jean; Maude Hamilton; Molly Sedeuil; Alexis Gonneaud; Line Berthiaume; Étienne Audet-Walsh; Véronique Giroux
Article Type: Research Article

Dear Dr Giroux,

I am happy to tell you that your manuscript has been accepted for publication in Journal of Cell Science, pending standard publication integrity checks.

© 2026. Published by The Company of Biologists under the terms of the Creative Commons Attribution License (https://creativecommons.org/licenses/by/4.0/).
